# *Selaginella brachyclada* reinstated from synonymy under *S. rhodostachya* (Lycopodiopsida, Selaginellaceae), with updated descriptions of both taxa

**DOI:** 10.3897/phytokeys.274.190998

**Published:** 2026-05-18

**Authors:** Iván A. Valdespino, Christian A. López

**Affiliations:** 1 Departamento de Botánica, Facultad de Ciencias Naturales, Exactas y Tecnología, Universidad de Panamá, Apartado Postal 0824–00073, Panamá, Panama Facultad de Ciencias Naturales, Exactas y Tecnología, Universidad de Panamá Panamá Panama; 2 Sistema Nacional de Investigación (SNI), SENACYT, Panamá, Panama Department of Integrative Biology, University of Texas at Austin Austin United States of America; 3 Smithsonian Tropical Research Institute, Tupper Building – 401, Roosevelt Ave., Panamá 0843–03092, Panama Smithsonian Tropical Research Institute Panamá Panama; 4 Department of Integrative Biology, University of Texas at Austin, 2415 Speedway #C0930, Austin, Texas 78712, USA Sistema Nacional de Investigación (SNI), SENACYT Panamá Panama

**Keywords:** Chartaceous, coriaceous, flagelliform, midribs, stomata, teeth-like, Cartáceo, coriáceo, estomas, flageliforme, nervaduras centrales, similar a dientes

## Abstract

*Selaginella brachyclada* is reinstated as a separate species, distinct from *S. rhodostachya*, based on a comparative morphological review of herbarium specimens, including type specimens. We provide updated descriptions and illustrations for both taxa and clarify their diagnostic features using macromorphological and micromorphological data from scanning electron microscopy (leaf and spore surface). We discuss differences between these taxa and other morphologically similar species to help ensure accurate identification and stabilize the use of names within the *Selaginella valdepilosa* group. Distributional and ecological information on *S. brachyclada* and *S. rhodostachya*, gathered from the examined specimens, is compiled and analyzed to support preliminary conservation assessments.

## Abstract (es)

*Selaginella brachyclada* se reclasifica como especie independiente, distinta de *S. rhodostachya*, luego de una revisión morfológica comparativa de especímenes de herbarios, incluyendo especímenes tipo. Proporcionamos descripciones e ilustraciones actualizadas de ambos taxones y aclaramos sus características diagnósticas con base en datos macromorfológicos y micromorfológicos obtenidos mediante microscopía electrónica de barrido (superficie de hojas y esporas). Analizamos las diferencias entre estos taxones y otras especies morfológicamente similares para lograr una identificación precisa y estabilizar el uso de los nombres dentro del grupo de *Selaginella valdepilosa*. Se recopila y analiza información distribucional y ecológica de *S. brachyclada* y *S. rhodostachya*, obtenida de los especímenes examinados, para respaldar las evaluaciones preliminares de conservación.

## Introduction

The *Selaginella valdepilosa* species group, originally termed the *Selaginella microdonta* group by Valdespino (2020), consists of a set of morphologically similar taxa, including *S. brachyclada* Baker, *S. breweriana* A.R.Sm., *S. cardiophylla* Valdespino, *S. cyclophylla* A.R.Sm., *S. hemicardia* Valdespino, *S. microdonta* A.C.Sm., *S. neblinae* A.R.Sm., *S. rhodostachya* Baker, *S. rostrata* Valdespino, and *S. valdepilosa* Baker. As here defined, this group shares a suite of morphological characters that include a ribbon-like or leafy liverwort- to moss-like habit, creeping stems, filiform rhizophores, small leaves, usually ovate, ovate-oblong, ovate-elliptic, and elliptic lateral leaves, and light yellow, yellow, or cream-colored megaspores (see Valdespino 2020). These morphological features often overlap among species. As a result, the boundaries among some species within the group have historically been unstable, and some authors have interpreted certain taxa differently, leading to confusion in taxonomic identification and nomenclature. One of the most persistent issues concerns the circumscription of *S. rhodostachya* and the status of *S. brachyclada*, which has often been considered a synonym of the former.

*Selaginella brachyclada* (Baker, 1887a: 45) was described from a collection made in Guyana in May 1887 (i.e., *G.S. Jenman 1481*). The same gathering was later used by Jenman (1887: 99) to describe *S. diminutifolia* Jenman on July 23, 1887. Therefore, the earlier species name has priority over the later epithet. By contrast, *S. rhodostachya* was described earlier by Baker (1886: 221) based on a collection from the Venezuelan side of Mount Roraima (i.e., [*E.F*.]. *im Thurn s.n*.).

*Selaginella brachyclada* and *S. rhodostachya* have long been misunderstood, possibly due to the limited number of specimens collected for these taxa and the lack of study of type material. As a result, *S. brachyclada* and *S. diminutifolia* were synonymized under *S. porelloides* (Lam.) Spring by Reed (1965[–1966]: 70 and 101, respectively). Vareschi (1969) recognized *S. brachyclada* as a valid species; however, his species description and drawings probably refer to a different taxon. Alston et al. (1981: 296) listed *S. brachyclada*, *S. brevispicata* Hieron. ex Bautista, and *S. diminutifolia* as synonyms of *S. rhodostachya*, and Mostacero (2008) adopted this interpretation.

Additionally, confusion has been worsened by the incorrect citation of the type of *S. rhodostachya*. Alston et al. (1981: 296) cited the type as “Guyana, Essequibo: foot of the Kaieteur Falls [*G.S*.] *Jenman 148*,” when the actual type locality is from Venezuela, as mentioned above. Furthermore, Jermy and Rankin in Alston et al. (1981: 292) described another taxon, *S. valdepilosa* subsp. *tricholoma* Jermy & J.M.Rankin, to include a plant collected by J.A. Steyermark from Mt. Roraima in Venezuela (i.e., *J.A. Steyermark 58016* [*58916*]), which was also mistakenly localized by the authors as originating from “Guyana. Essequibo: vertice montis Roraima.” According to Jermy and Rankin in Alston et al. (1981: 292), the later taxon “resembled *S. rhodostachya* (No. 86), which has more ovate lateral leaves,” but they considered it “very close to *S. valdepilosa* [Baker] s.str.,” and suggested that “eventually it may prove to be a growth form of the latter.”

Valdespino (1992: 202) noted that *S. rhodostachya* was an ill-defined species that included at least three distinct taxa. Smith (1995) merged *S. brevispicata* and *S. valdepilosa* subsp. *tricholoma* under *S. rhodostachya*, but did not consider *S. brachyclada*. Cremers et al. (2007) treated *S. brachyclada*, *S. brevispicata*, and *S. diminutifolia* as synonyms of *S. rhodostachya*. Mostacero (2008: 169) agreed with previous authors in treating *S. brachyclada* and *S. brevispicata* as synonyms of *S. rhodostachya* but ignored *S. diminutifolia*. More recently, Góes-Neto et al. (2017) further misinterpreted *S. rhodostachya*, by considering it a polymorphic species that may constitute a species complex, and compared it with *S. microdonta*.

Much of the intensive botanical exploration of the tepuis of the Guayana Highlands on the Venezuelan side took place during the mid- to late twentieth century (particularly from the 1950s through to the 1990s). This resulted in numerous collections of *Selaginella*, as well as other plant groups. These include many specimens often identified as *S. rhodostachya*. A closer comparison with type material suggests that some of those specimens align more closely with *S. brachyclada* than with *S. rhodostachya* s.str. Accordingly, a reassessment of the circumscription, species boundaries, and synonymy of both taxa is warranted, as detailed below.

### Methodology

Detailed revision of herbarium specimens, including examination of type material and digital images from B, BM, F, G, GH, K, L, MG, MICH, MO, NY, P, U, UC, and US (codes follow Thiers 2026), as well as consultation of specimens from the PteridoPortal (2026), was conducted. Additionally, scanning electron microscopy (SEM) was used to analyze leaf and spore characters. The assessment of species conservation status was conducted in accordance with the IUCN Standards and Petitions Committee (2022) guidelines, considering threats and estimating the extent of occurrence (EOO) and area of occupancy (AOO) using GeoCAT (Bachman et al. 2011), which is continuously updated at https://geocat.kew.org/.

## Results

Our examination of the types of *S. brachyclada*, *S. brevispicata*, *S. diminutifolia*, *S. rhodostachya*, *S. valdepilosa*, and *S. valdepilosa* subsp. *tricholoma* clarified the distinctions among species within the *Selaginella valdepilosa* group. This was supported by the study of additional collections from the Guayana Highlands and Venezuelan tepuis, herbarium images from the PteridoPortal (2026), and SEM analyses of leaf and spore surfaces. Overall, these data enabled us to revisit and resolve the long-standing confusion between *S. brachyclada* and *S. rhodostachya*. Our study supports the reinstatement of *S. brachyclada* as a species distinct from *S. rhodostachya* and provides a more precise circumscription for *S. rhodostachya* s.str. Within this revised framework, we refine the synonymy associated with *S. rhodostachya*: *S. brevispicata* and *S. valdepilosa* subsp. *tricholoma* are retained as conspecific with *S. rhodostachya*, whereas *S. brachyclada* and *S. diminutifolia* are excluded. *Selaginella brachyclada* is here recognized as a distinct species, and *S. diminutifolia* is confirmed as conspecific with, and therefore a synonym of, said species. By contrast, *S. microdonta* is not closely related to *S. brachyclada*, as suggested by (Góes-Neto et al. 2017), but rather to *S. cardiophylla*, *S. hemicardia*, and *S. rostrata*, among other species of the *Selaginella microdonta* group (Valdespino 1992, 2020), which, as mentioned above, is here renamed the *Selaginella valdepilosa* group. Finally, we correct the collector number of the type collection of *S. valdepilosa* subsp. *tricholoma*, which should be cited as *J.A. Steyermark 58916* rather than *J.A. Steyermark 58016*.

### Taxonomic treatment

#### Selaginella brachyclada

Taxon classification

Plantae

SelaginellalesSelaginellaceae

Baker

0B9380DD-73C0-549F-AD96-6A921C072B6E

Figs 1, 2

Selaginella brachyclada Baker, Fern Allies: 45. 1887 [publ. May. 1887] ≡ Selaginella diminutifolia Jenman, Gard. Chron., ser. 3. 2: 99. 1887 [pub. 23 Jul. 1887]. Type: Guyana. Essequibo, [Potaro River], foot of the Kaieteur, [5°13'N, 59°25'W], [Sep. – Oct. 1881], *G.S. Jenman 1481* (holotype: K! [K000589210]; isotypes: BM! [BM000905721], NY!).

##### Description.

***Plants*** terrestrial or epipetric. ***Stems*** creeping, stramineous, 3–20 cm long, 0.2–0.6 mm diam., non-articulate, not flagelliform or stoloniferous, 2–3-branched, these often flagelliform. ***Rhizophores*** ventral, borne throughout the stem length, filiform, 0.1–0.3 mm diam. ***Leaves*** chartaceous or coriaceous, heteromorphic, of three types (ventral, median, and axillary). ***Lateral leaves*** distant, ascending and spreading at 60° to the stems, ovate-deltate on main stems or narrowly ovate to ovate, or ovate-oblong on distal portions of the main stems and branches, 1.3–2.5 × 1.0–1.9 mm; bases subcordate on main stems or rounded on distal portions of main stems and branches, glabrous, the acroscopic bases strongly overlapping stems, the basiscopic bases free from the stems; acroscopic and basiscopic margins greenish, continuously bordered by quadrangular cells, sparsely denticulate or entire throughout, the acroscopic and basiscopic margins on the lower surfaces hyaline or greenish hyaline, bordered by a band 1–5 cells wide of elongate, straight-walled and papillate idioblasts, the papillae 4–12 in one row in each cell lumen; apices acute to broadly acute, tipped by 2–5, short, tooth-like hairs; upper surfaces glabrous, except for submarginal tooth-like hairs along the basiscopic margins, comprising rounded to quadrangular, irregularly, sinuate-walled and papillate cells, the papillae 2–6 on each cell lumen, with stomata along the basiscopic margins, and with submarginal tooth-like hairs, the lower surfaces comprising elongate, sinuate-walled, and laevigate cells and with some, sparsely distributed, elongate, sinuate-walled, and papillate idioblasts, the papillae 15–35 arranged in two rows on each cell lumen, with stomata in 1–3 rows along midribs and on 1 row along basiscopic margins, the midribs stramineous and plane on side view. ***Median leaves*** distant, ascending and overlapping the stems, ovate to broadly ovate, or ovate-deltate to cordate, 0.8–1.5 × 0.5–1.2 mm, bases glabrous, rounded or subcordate, without auricles; the inner margins on both surfaces hyaline, continuously bordered by a narrow band of idioblasts, the band 1–3 cells wide, the idioblasts as in the lateral leaves, denticulate or entire, the outer margins on both surfaces greenish, comprised of quadrangular cells along proximal ^1^/_2_, and distally as in the inner margins; apices broadly acute or shortly attenuate, each usually tipped by 1–4 tooth-like, stiff, short hairs or this caducous; the upper surfaces glabrous, comprised of quadrangular to rounded, papillate cells, the papillae 4–6 on each cells lumen, without idioblasts, with the midribs prominently raised on side view along distal ^1^/_2_–^3^/_4_ where stomata are found in 1–4 rows and submarginally on the outer margins, the lower surfaces as in the lateral leaves. ***Axillary leaves*** ovate-deltate to subcordate or ovate-lanceolate, 1.2–2.5 × 0.8–1.8 mm; bases subcordate to rounded; margins, apices, and leaf surfaces as in the lateral leaves. ***Strobili*** terminal on the branch tips, laxly quadrangular to flattened and dorsiventral, 10–15 mm. ***Sporophylls*** monomorphic, ascending, imbricate, keeled along the midribs, the keels distally puberulent with short, teeth-like projections on the upper surfaces, without laminar flaps, ovate, 0.8–1.0 × 0.5–0.8 mm; bases rounded to rounded-oblique; apices acute to shortly-attenuate, 0.05–0.1 mm long; stomata found along the midribs; ***dorsal sporophylls*** with the inner margins hyaline, bordered by 1–3 rows of elongate, straight-walled and papillate idioblasts similar to those of the median leaf margins, without marginal stomata, entire on proximal ^1^/_2_–^2^/_3_, otherwise sparsely denticulate distally, the outer margins greenish, bordered by rounded to quadrangular cells and with marginal stomata along proximal ^1^/_2_, entire along proximal ^1^/_2_, otherwise sparsely denticulate distally; the upper and lower surfaces of the leaf laminae as in the median leaf surfaces; ***ventral sporophylls*** margins hyaline, bordered by 1–3 rows of elongate, straight-walled and papillate idioblasts, entire on proximal ^1^/_4_ and sparsely denticulate along distal ^3^/_4_, without marginal stomata; apices as in the dorsal sporophylls; the upper and lower surfaces of the leaf laminae hyaline to greenish-hyaline, composed of elongate, straight-walled, and laevigate cells. ***Megasporangia*** in two ventral rows; ***megaspores*** light yellow to cream, with a prominent equatorial flange, proximal faces rugulate to rugulate-reticulate, distal faces reticulate, the microstructure of proximal and distal faces not observed, 350–400 µm diam. ***Microsporangia*** in two dorsal rows; ***microspores*** orange, proximal and distal faces laevigate, micro-ornamentation not observed, 25–48 µm diam.

**Figure 1.**
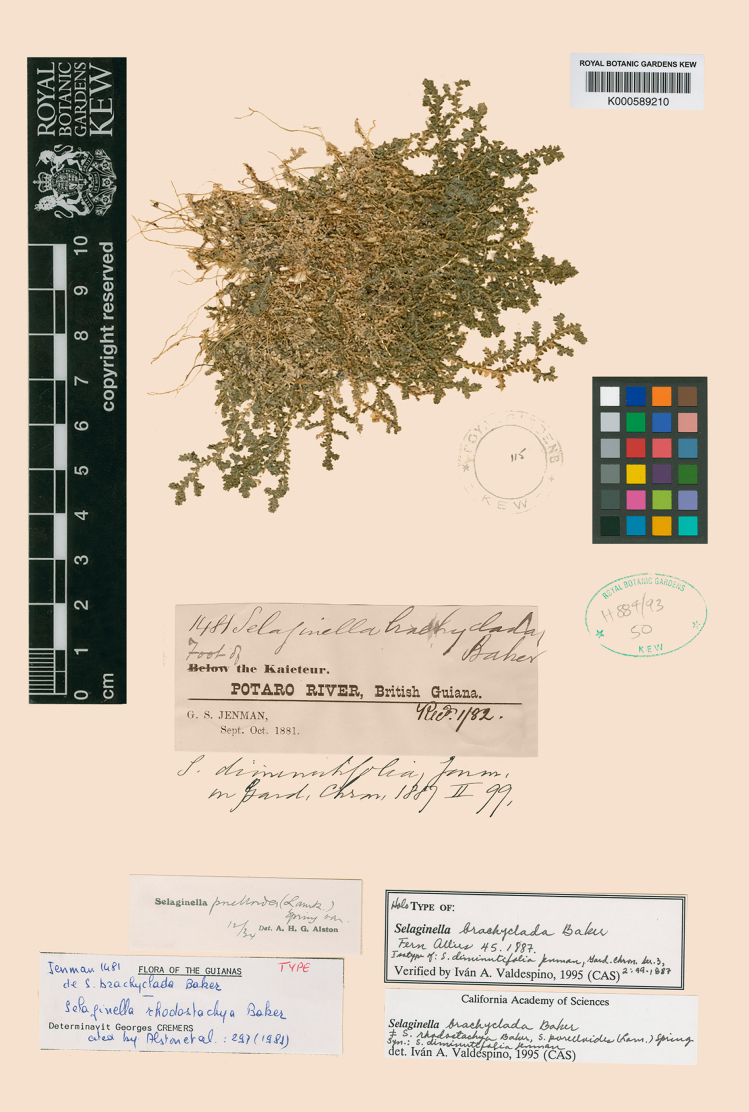
Holotype of *Selaginella brachyclada*. Digitized image, courtesy of the Royal Botanic Gardens, Kew (K).

**Figure 2.**
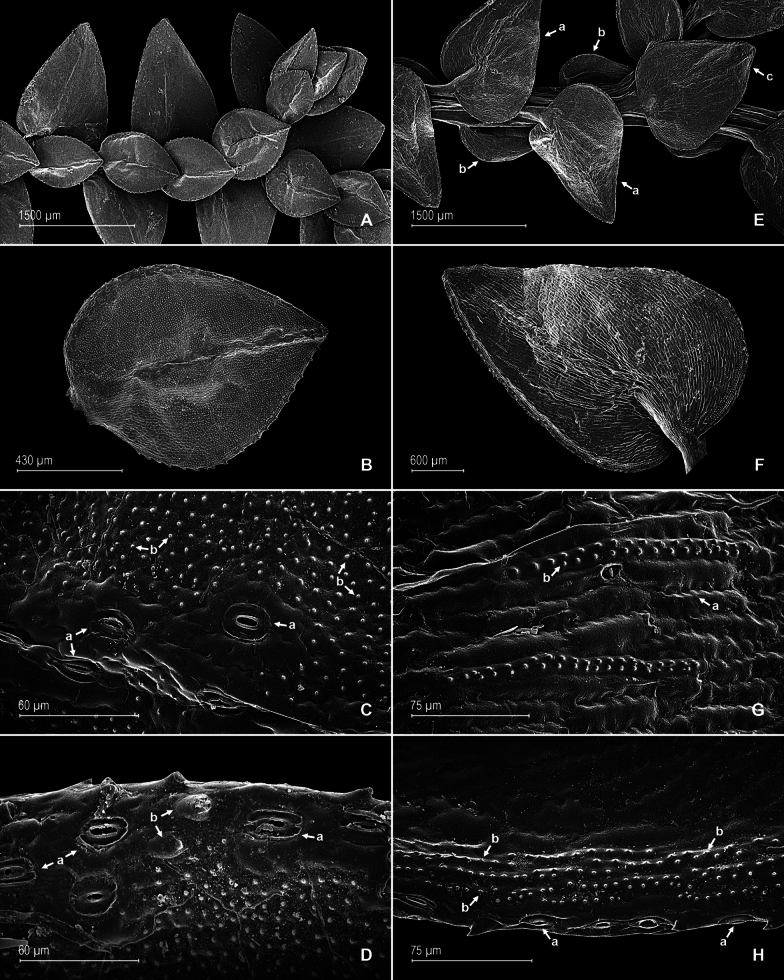
*Selaginella brachyclada*. **A**. Section of the stem, upper surface; **B**. Median leaf, upper surface; **C**. Close-up of median leaf mid-section, upper surface; note stomata along the midrib (a) and papillate epidermal cells (b); **D**. Lateral leaf basiscopic margin, upper surface; note stomata (a) and tooth-like hairs (b); **E**. Section of the stem, lower surface; note lateral (a), median (b), and axillary (c) leaves; **F**. Lateral leaf, lower surface; **G**. Close-up of lateral leaf, lower surface; note elongate, sinuate-walled, and laevigate cells (a) and elongate, straight-walled, and papillate idioblasts (b); **H**. Close-up of a section of the basiscopic margin of the lateral leaf, lower surface; note marginal stomata (a) and elongate, straight-walled, and papillate idioblasts (b). (**A**–**H**) from *J.A. Steyermark et al. 115583*, GH.

##### Habitat and distribution.

*Selaginella brachyclada* typically grows on moist rock ledges and seepage surfaces near waterfalls in tropical savanna and along gallery and riparian forests. It is known from Guyana (the type locality) and, in Venezuela, it occurs at mid-elevations (995–1200 m) in the Gran Sabana region (Guayanan savanna ecoregion), Bolívar State, southeastern Venezuela. Due to the continuity of suitable habitats, the species is likely to occur in neighboring Roraima, in northern Brazil, although this has not yet been confirmed.

##### Conservation status.

*Selaginella brachyclada* is known from only six collections: the type, collected at the base of Kaieteur Falls in Kaieteur National Park (Guyana), and five collections from the Gran Sabana region within Canaima National Park (Bolívar State, Venezuela). Using GeoCAT with the IUCN-recommended 2-km grid, these records yield an extent of occurrence (EOO) of 4,128.942 km^2^ and an area of occupancy (AOO) of 20 km^2^. Although *Selaginella brachyclada* is known from protected areas (e.g., Canaima National Park in Venezuela), portions of the Gran Sabana sector are affected by frequent, human-ignited fires linked to local land-use practices such as shifting cultivation and hunting (Bilbao et al. 2010). Changes in fire use have been associated with forest and habitat loss, and fire is considered a major threat because it may promote forest substitution by treeless savannas in the savanna–forest mosaic of the Gran Sabana (Bilbao et al. 2010). In addition, gold mining within Canaima National Park has been documented as largely informal and illegal, often conducted without permits, supporting its classification as an ongoing pressure that can drive local habitat disturbance and degradation (García Bonet 2020). The species is therefore assessed as Endangered under IUCN Criterion B (EN B1ab(iii)+B2ab(iii)) following IUCN Standards and Petitions Committee (2022).

##### Specimens examined.

**Venezuela** • Bolívar: 75 km N of Santa Elena de Uairén and 232 km S of El Dorado, 5°15'N, 61°15'W, alt.1200 m, 19 Dec. 1978, *J.A. Steyermark & V. Carreño E. 117881* (GH, VEN), *J.A. Steyermark & V. Carreño E. 117886* (GH, VEN); • Gran Sabana, near Salto del Aponguao, 42.5 km NE of Misión of Santa Teresita de Kavanayén, alt. 1130 m, 22 Feb. 1978, *J.A. Steyermark et al. 115575* (BM, GH, VEN), *J.A. Steyermark et al. 115583* (BM, GH, VEN); • Kama-Merú, alt. 995 m, 6 Apr. 1988, *V. Marcano, C. Sastre, F. Sastre & S. Hernández 999* (mixed coll. *999a* at P, VEN).

##### Discussion.

*Selaginella brachyclada* is characterized by its ovate to broadly ovate, or ovate-deltate to cordate median leaves with rounded to subcordate bases, with the inner margins hyaline and denticulate or entire, the apices broadly acute or shortly attenuate, each usually tipped by 1–4 stiff, tooth-like, short hairs or these caducous, with the midribs prominently raised on side view along distal ^1^/_2_–^3^/_4_ where stomata are arranged in 1–4 rows and submarginally on the outer margins. Additionally, *S. brachyclada* has distinctly ovate-deltate to subcordate or ovate-lanceolate axillary leaves with subcordate to rounded bases and denticulate margins.

As noted earlier, *Selaginella brachyclada* was historically misidentified as *S. porelloides* and was later synonymized with *S. rhodostachya*. It is distinguished from *S. porelloides* by its ovate-deltate (vs. semicordiform to cordiform) lateral leaves with acroscopic margins greenish (vs. hyaline), denticulate (vs. serrate to shortly ciliate along proximal ^1^/_2_, serrulate to entire distally), and median leaves with rounded to subcordate (vs. cordate to subcordate) bases, lacking (vs. with two slightly developed or less frequently lacking) auricles, broadly acute or shortly attenuate (vs. short- to long-acuminate or less frequently short-aristate) apices, the tip less that ^1^/_8_ (vs. acumen or arista ^1^/_4_ or less) the length of laminae, each 0.1 mm or less (vs. 0.3–0.6 mm) long. Additionally, the median leaf upper surfaces have raised (vs. plane) midribs with inconspicuous stomata along the distal ^1^/_2_–^3^/_4_ and submarginally on the outer margins (vs. with conspicuous stomata scattered throughout the leaf laminae).

*Selaginella brachyclada* differs from typical *S. rhodostachya* (i.e., those specimens with caducous, leaf marginal hairs) by its ovate to broadly ovate or ovate-deltate to cordate (vs. ovate-elliptic) median leaves, usually tipped by 1–4 tooth-like, stiff, and short (vs. two, divergent, long) hairs, and ovate-deltate (vs. narrowly-elliptic or elliptic-ovate) lateral leaves. It’s lateral leaves with subcordate (vs. rounded) bases on the main stems, and ovate-deltate to subcordate or ovate-lanceolate (narrowly elliptic) axillary leaves. *Selaginella brachyclada* is even more clearly distinguished from the form of *S. rhodostachya* formerly assigned to *S. valdepilosa* Baker subsp. *tricholoma* by its sparsely denticulate (vs. long-ciliate) leaf margins.

*Selaginella brachyclada* may be confused with *S. muscosa* Spring, from which it differs by its coriaceous (vs. papyraceous) leaves, denticulate (vs. shortly ciliate along basal ^3^/_4_, otherwise serrate to serrulate along distal ^1^/_4_) median leaf margins with acute to short-acuminate (vs. short to long-acuminate or long-aristate) apices, and by lacking (vs. with conspicuous) idioblasts on the upper leaf surfaces.

#### Selaginella rhodostachya

Taxon classification

Plantae

SelaginellalesSelaginellaceae

Baker

0BE5AD3C-4E9E-5EEF-9CFE-BF0D9605161E

**Figure 3.**
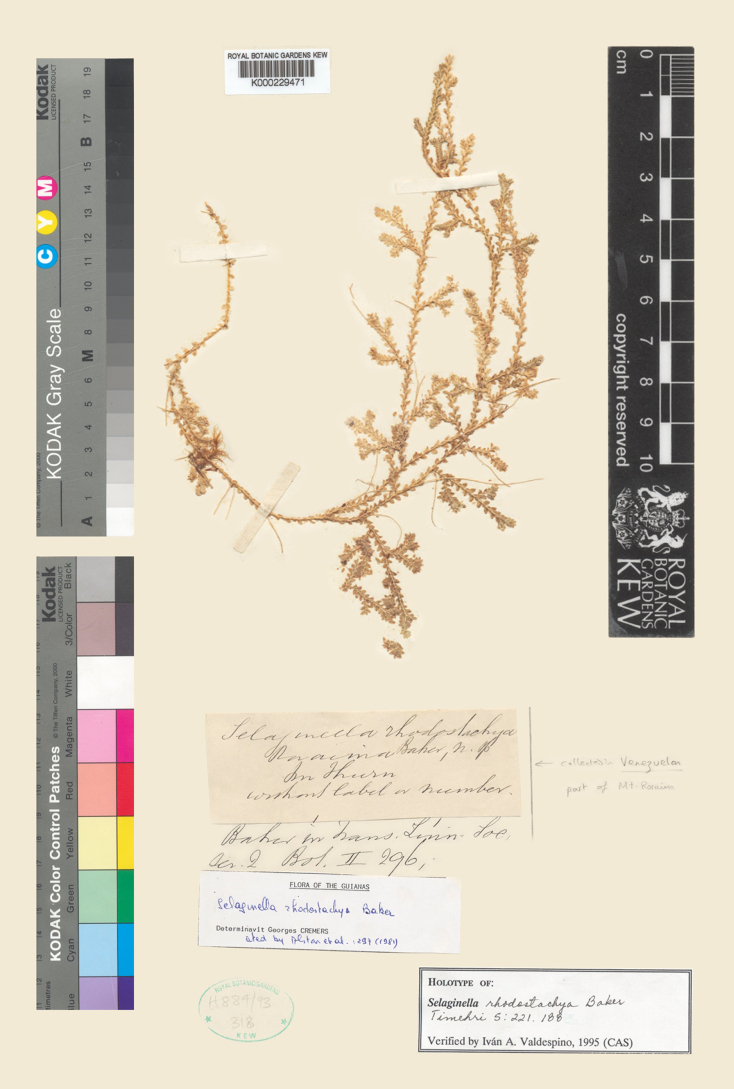
A holotype of *Selaginella rhodostachya*. Digitized image, courtesy of the Royal Botanic Gardens, Kew (K); the elements reoriented.

**Figure 4.**
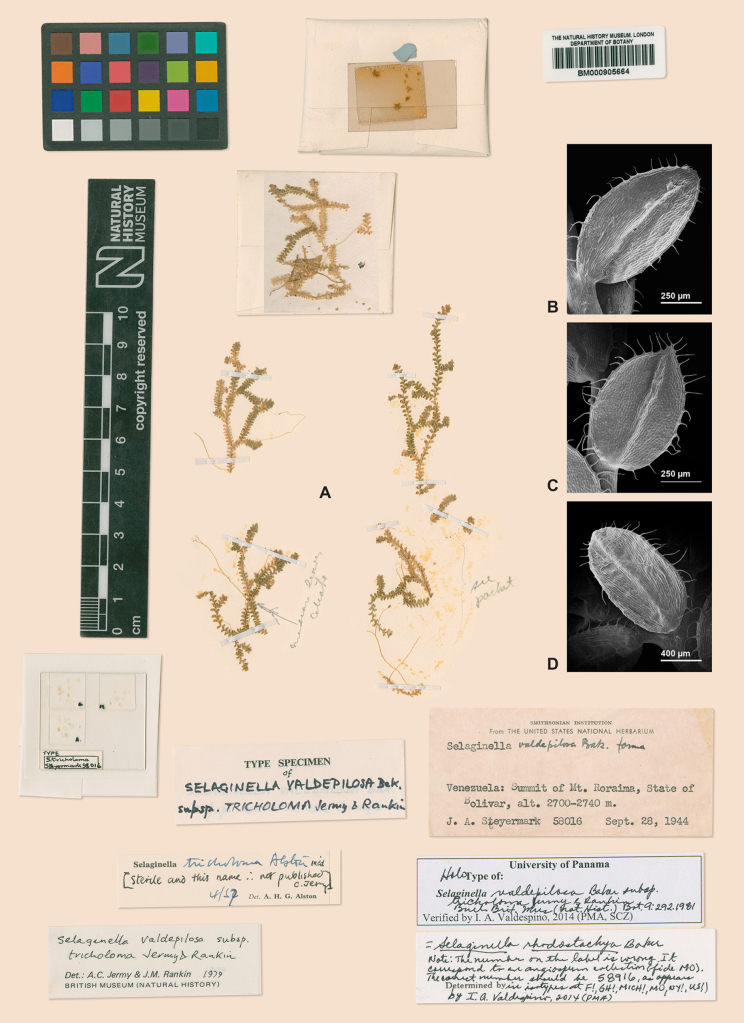
A holotype of *Selaginella valdepilosa* subsp. *tricholoma* = *S. rhodostachya*. **A**. Plant material; note creeping stems; **B**. Lateral leaf, lower surface; note long-ciliate margins and prominent midrib; **C**. Median leaf, upper surface; note long-ciliate margins, prominent midrib, and acute apex tipped by two divergent long-cilia; **D**. Axillary leaf, lower surface; note margins as in (**A**) and (**B**). (**A**–**D**) from *J.A. Steyermark 58016* [*58916*], BM. Digitized image, reorganized based on the holotype at the herbarium of The Natural History Museum (BM).

Selaginella rhodostachya Baker, Timehri 5: 221. 1887. Type: Guyana. [Venezuela]: Mt. Roraima, [Oct. 1884 to Jan. 1885], [*E.F*.] *im Thurn s.n*. (holotype: K! [K000229471]; isotypes: BM! [as *im Thurn 226*, an erroneous number; most likely *im Thurn s.n*., BM014638426], US! [as *im Thurn 271*, an erroneous number; most likely *im Thurn s.n*., US00135738]. Fig. 3.
= Selaginella brevispicata Hieron. ex Bautista, Acta Amazonica 4: 19. 1974. Type: Brazil. Roraima: [Mt.] Roraima, alt. 2300 m, Dec. 1909, *E. Ule 8491* (holotype: MG-digital image! [MG013555]; isotypes: B!-2 sheets [B 20 0095099 & B 20 0095100], BM! [BM000905714], G! [G00349430], K! [K000589268], L! [L 0057418]).
= Selaginella valdepilosa Baker subsp. *tricholoma* Jermy & Rankin, Bull. Brit. Mus. (Nat. Hist.), Bot. 9: 292. 1981. Type: Venezuela: Bolívar, summit of Mt. Roraima, [on southern half of the summit between Summit Camp, Central Rift, Central Swamp, and pond at southern end], alt. 2700–2740 m, 28 Sep. 1944, *J.A. Steyermark 58916* (holotype: BM! [as *58016*] [BM000905664]; isotypes: F! [Herb. No. 1208244], G!, GH p.p.!, L!, MICH! [MICH1191442], MO! [MO-255519], NY! [NY00020658], US-2 sheets! [Herb. Nos. 1915037 & 1915105]). Fig. 4.

##### Description.

***Plants*** terrestrial or epipetric. ***Stems*** creeping, stramineous, 10–15 cm long, 0.2–0.5 mm diam., non-articulate, not flagelliform or stoloniferous, 1–3-branched, determinate. ***Rhizophores*** dorsal, ventro-axillary, or axillary, borne throughout the stem length, filiform, 0.1 or 0.2 mm diam. ***Leaves*** chartaceous to coriaceous, heteromorphic, of three types (ventral, median, and axillary). ***Lateral leaves*** distant, ascending and spreading at 45° to the stems, elliptic to elliptic-ovate, 0.7–2.0 × 0.5–1.2 mm; bases rounded, glabrous, the acroscopic bases strongly overlapping stems, the basiscopic bases free from the stems; acroscopic and basiscopic margins on the upper and lower surfaces hyaline or greenish-hyaline, continuously bordered by a band 1–3 cells wide of idioblasts, the idioblasts elongate, straight-walled and papillate, the papillae in a single row on each cell lumen, entire throughout or entire along proximal ^3^/_4_ and scarcely denticulate along distal ^1^/_4_, or long-ciliate throughout; apices obtuse, tipped by 6–8, short, tooth-like hairs; upper surfaces glabrous, comprised of rounded, sinuate-walled, and papillate cells, the papillae 2–6 on each cell lumen, without stomata, the lower surfaces comprising elongate, sinuate-walled, and laevigate cells and with some, sparsely distributed, elongate, sinuate-walled, and papillate idioblasts, the papillae 10–20 arranged in a single row on each cell lumen, with stomata along the distal ^1^/_2_ of the midribs in 1–3 rows along the midribs, without submarginal stomata or tooth-like hairs, the midribs straw-colored and prominently raised on side view. ***Median leaves*** distant or imbricate towards stem and branch apices, ascending and spreading at 70° to the stems, elliptic, elliptic-ovate, or broadly ovate, 0.9–1.4 × 0.5–1.0 mm; bases glabrous, rounded, without auricles; margins on the upper and lower surfaces hyaline, continuously bordered by a narrow band of idioblasts, the band 1 or 2 cells wide, the idioblasts as in the lateral leaves, entire throughout or entire along the proximal ^3^/_4_ and scarcely denticulate along the distal ^1^/_4_, or long-ciliate throughout; apices acute, broadly acute to obtuse, each tipped by 1–5 tooth-like, projections, often caducous, or by 2 divergent, long cilia; upper surfaces glabrous, comprise of rounded, sinuate-walled, and papillate cells, the papillae 2–6 on each cell lumen, without stomata, the lower surfaces comprising elongate, sinuate-walled, and laevigate cells and with some, sparsely distributed, elongate, sinuate-walled, and papillate idioblasts, the papillae 10–20 arranged in a single row on each cell lumen, the midribs green and prominently raised on side view throughout the leaf length or along distal ^1^/_2_ where the stomata are present along the distal ^1^/_2_ of the midribs in 1 or 3 rows, without submarginal stomata or tooth-like hairs. ***Axillary leaves*** elliptic or narrowly ovate to ovate-lanceolate, 1.0–1.4 × 0.5–0.8 mm; bases rounded; margins, apices, and leaf surfaces as in the lateral leaves. ***Strobili*** terminal on the branch tips, loosely quadrangular, 2–5 mm long. ***Sporophylls*** monomorphic, ascending, imbricate, keeled along the midribs, the keels glabrous on the upper surfaces, without laminar flaps, ovate to ovate-elliptic, broadly elliptic or broadly ovate, 1.0–1.4 × 0.6–0.8 mm; bases rounded; apices acute or obtuse, tipped by 2–5 short, short-teeth-like projections or 2 divergent, long-cilia; stomata found along the midribs; ***dorsal sporophylls*** with margins hyaline, bordered by 1 or 2 rows of elongate, straight-walled and papillate idioblasts similar to those of the median leaf margins, without marginal stomata, entire throughout or entire on proximal ^2^/_3_, otherwise sparsely denticulate distally; upper surfaces greenish, comprised of rounded to quadrangular cells, except for the leaf half that imbricate with the ventral sporophylls, where the cells are elongate and straight-walled, the lower surfaces comprised of elongate and straight-walled cells; ***ventral sporophylls*** with margins hyaline similarly bordered as in the dorsal sporophylls, entire on proximal ^3^/_4_ and sparsely denticulate along the distal ^1^/_4_, without marginal stomata; the upper and lower surfaces hyaline, composed of elongate, straight-walled and laevigate cells. ***Megasporangia*** in two ventral rows; ***megaspores*** light yellow, yellow, or cream, without an equatorial flange, proximal and distal faces psilate to slightly rugulate, the microstructure of proximal and distal faces not observed, 200–300 µm diam. ***Microsporangia*** in two dorsal rows; ***microspores*** orange, proximal and distal faces psilate, micro-ornamentation not observed, 20–40 µm diam.

##### Habitat and distribution.

*Selaginella rhodostachya* is found on muddy soils, in rock crevices, and at the bases of wet bluffs within shrubland and forest at 1,350–2,800 m. It is a typical species of the Guayana Highlands, recorded from the Mazaruni-Potaro region of Guyana, the Amazonas and Bolívar states of Venezuela, and Roraima in Brazil.

##### Conservation status.

*Selaginella rhodostachya* is known from multiple localities in the Guayana Highlands, with confirmed collections from Venezuela, Guyana, and Brazil. The available georeferenced records indicate an extent of occurrence (EOO) of 21,005.349 km^2^ and an area of occupancy (AOO) of 28 km^2^. According to the IUCN Standards and Petitions Committee (2022) guidelines, we recommend a preliminary conservation status of Vulnerable (VU B2ab(iii)), due to its limited AOO and occurrence at a few locations. Additionally, ongoing expansion of extractive activities, particularly gold mining, along with logging, agriculture, and infrastructure development in the Guiana Shield, suggests a continuing decline in habitat quality in parts of the Guayana Highlands (Bovolo et al. 2018).

##### Specimens examined.

**Guyana** • [**Cuyuni-Mazaruni**]: slopes near Roraima, *G.S. Jenman s.n*. (NY). **Venezuela** • **Amazonas**: Río Negro, Cerro Aracamuni summit, Proa Camp, 01°32'N, 65°49'W, alt. 1400 m, 30 Oct. 1987, *R.L. Liesner & G. Carnevali 22659* (U-digital image, VEN-digital image), • Popa Camp, 01°26'N, 65°47'W, alt. 1550 m, 18 Oct. 1987, *R.L. Liesner & F. Delascio 22129* (U-digital image, VEN-digital image); • Camp XI, Cerro de la Neblina, 6.2 km NNE, Pico Phelps (= Neblina), 20.5 km ENE Neblina Base Camp, 00°51'45"N, 65°58'52"W, alt. 1350–1450 m, 26 Feb. 1985, *J.M. Beitel 85272* (NY, VEN-digital image). • **Bolívar**: Kukenan Tepuy, alt. 2600 m, Jan. 1977, *F. Delascio & C. Brewer 4937* (MO, UC, US), 05°13'N, 60°18'W, alt. 2550 m, 11 Apr. 1988, *R.L. Liesner 23111* (MO, UC, US, VEN), 12 Apr. 1988, *R.L. Liesner 23181* (MO, NY, U, UC, VEN); • Mt. Roraima, ledge above Rondon Camp, alt. 6900 ft [2103.12 m], Dec. 1884, *im Thurn 226* (BM), ledge above Rondon Camp, alt. 6900 ft [2103.12 m], 1 Dec. 1927, *G.H.H. Tate 466* (BM, *466b* at NY), alt. 2255–2620 m, 27 Sep. 1944, *J.A. Steyermark 58753* (mixed coll. *58753a* at F, mixed coll. *58753a* at GH, MO, NY, US), 05°12'N, 60°42'W, alt. 2750–2800, 26 Aug. – 2 Sep. 1976, *J.A. Steyermark et al. 112530* (U), 26 Aug. 1976, *J.A. Steyermark et al. 112556* (NY, US, VEN-digital image), • Cima del Roraima-tepui, lower part of Valle de los Cristales, 05°11'58"N, 60°43'58.08"W, alt. 2680 m, 24 Mar. 2012, *Y. Vivas et al. 3077B* (UC, VEN). Roraima, Nov. – Dec. 1931, *N.J. Abbensetts 1A* (BM-2 sheets). **Brazil** • **Roraima**: Dec. 1909, *E. Ule 8491* (BM-text & illustration from original paper, MG), *E. Ule 8617* (B).

##### Discussion.

*Selaginella rhodostachya* is a distinctive species that has often been misunderstood. It is characterized by its elliptic to elliptic-ovate median leaves with distinctly keeled midribs on the upper surfaces, with stomata along the keel, narrowly hyaline margins comprised of one or two rows of papillate idioblasts in a single row. Its margins are entire throughout or entire along proximal ^3^/_4_ and scarcely denticulate along the distal ^1^/_4_, or long-ciliate throughout, with obtuse to rounded apices, each tipped by 1–5 tooth-like projections, often caducous, or 2 divergent, long cilia. Additionally, it has elliptic to elliptic-ovate lateral leaves with margins and apices similar to those of the median leaves, as well as elliptic or narrowly ovate to ovate-lanceolate axillary leaves, with margins and tips also resembling the median leaves. Furthermore, *S. rhodostachya* has dorsal, ventro-axillary, or axillary rhizophores, short strobili measuring 2–5 mm in length, composed of ovate to ovate-elliptic, broadly elliptic, or broadly ovate sporophylls, with midribs, margins, and tips similar to those of the median leaves. It also has microsporangia arranged in two dorsal rows, megasporangia along two ventral rows, and megaspores that are light yellow, yellow, or cream.

*Selaginella rhodostachya* has been mistaken for *S. brachyclada*, which was included under the former by some authors (e.g., Alston et al. 1981; Cremers et al. 2007; Mostacero, 2008). The features used to distinguish these two species are described under *S. brachyclada*.

**Figure 5.**
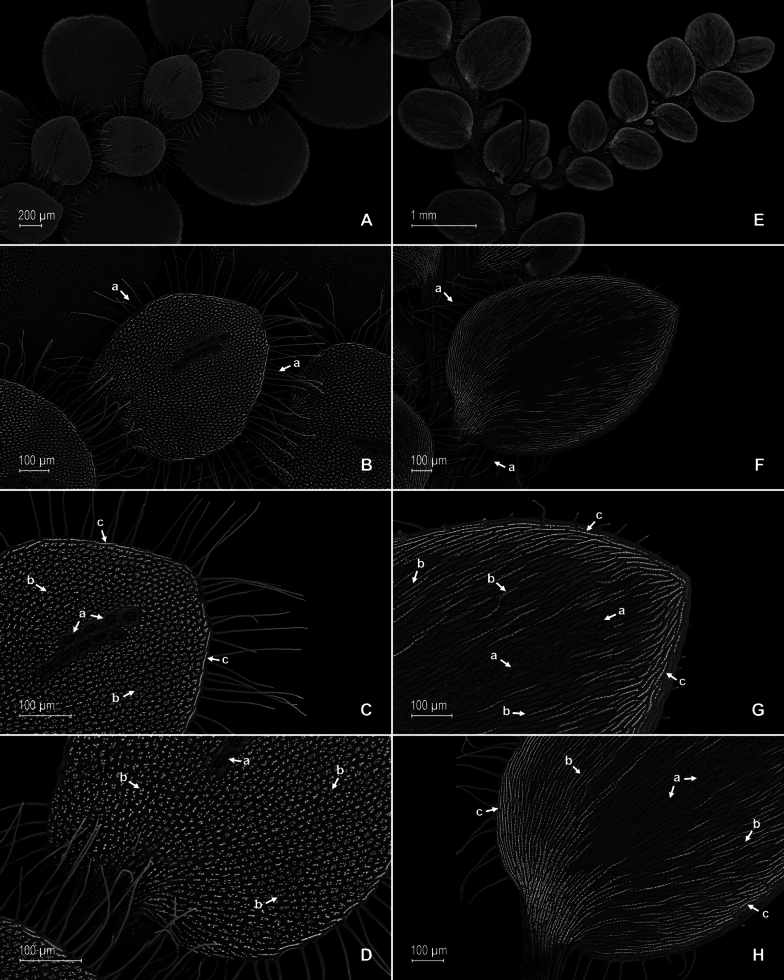
*Selaginella valdepilosa*. **A**. Section of the stem, upper surface; **B**. Median leaf, upper surface; note long-ciliate margins (a); **C**. Close-up of median leaf distal half, upper surface; note stomata (a), papillate epidermal cells (b), and marginal, elongate, straight-walled, and papillate idioblasts (c); **D**. Close-up of median leaf proximal half, upper surface; note stomata (a) and papillate, epidermal cells (b); **E**. Section of the stem, lower surface; **F**. Lateral leaf, lower surface; note marginal, long-cilia (a); **G**. Close-up of distal half of median leaf; note stomata (a), laminar (b) and marginal (c) elongate, straight-walled, and papillate idioblasts; **H**. Close-up of lateral leaf proximal half, lower surface; note stomata (a), laminar (b) and marginal (c), elongate, straight-walled, and papillate idioblasts. (**A**–**H**) from *R.L. Liesner 16733*, MO.

According to Jermy and Rankin (in Alston et al. 1981: 294), Alston was inclined to classify specimens with long-ciliate leaf margins and two divergent cilia at their tips as a distinct, new species previously identified as *S. rhodostachya*. However, Jermy and Rankin (in Alston et al. 1981: 292) considered these specimens more similar to *S. valdepilosa* (Fig. 5) and proposed the subspecies *S. valdepilosa* subsp. *tricholoma* to categorize them taxonomically. Additionally, in both *S. rhodostachya* and its segregate *S. valdepilosa* subsp. *tricholoma*, leaf marginal indument appears to be a variable morphological character. Some specimens, such as *Steyermark 58753* at GH!, that were collected near the same locality as the type of *S. valdepilosa* subsp. *tricholoma*, match the typical *S. rhodostachya*. These specimens lack cilia on their lateral and axillary leaf margins; however, their median leaves have short- to long-ciliate margins along the distal ^1^/_8_–^1^/_2_ of the leaf laminae, with apices tipped by 1 or 2 divergent long cilia, which are characteristic of *S. valdepilosa* subsp. *tricholoma*. Similarly, specimens from Kukenan Tepui, Bolívar State, Venezuela, have sparsely ciliate leaf margins but match the types of both *S. rhodostachya* and *S. valdepilosa* subsp. *tricholoma* in all other features. It appears that in *S. rhodostachya*, the marginal leaf indument is often caducous even within geographically close populations. Accordingly, we agree with Smith (1995) and Cremers et al. (2007), who synonymized *S. valdepilosa* subsp. *tricholoma* under *S. rhodostachya*.

Specimens of *S. rhodostachya* described as *S. valdepilosa* subsp. *tricholoma* share characteristics with *S. valdepilosa*, such as notably long-ciliate leaf margins with apices tipped by long, divergent cilia. Nonetheless, *S. valdepilosa* (Fig. 5) is distinguished from the former by its more delicate, liverwort-like growth form, broad ovate-orbicular to orbicular lateral leaves with the lower surfaces completely covered by long idioblasts, and long cilia mostly along the acroscopic leaf margins with few on the proximal ^1^/_4_, as well as ovate-orbicular to orbicular median leaves. In fact, *S. valdepilosa* is quite similar morphologically to *S. cyclophylla*, but it differs in having long-ciliate (vs. entire) leaves, and median leaves with keeled (vs. plane) midribs on the upper surfaces of the laminae, which have conspicuous stomata throughout their length in 1–4 rows (vs. stomata absent or not visible on upper leaf surfaces). Additionally, the lateral leaf lower surfaces are shiny with long, papillate idioblasts throughout (vs. leaf lower surfaces dull, made up of seemingly roundish or elongate cells without papillate idioblasts, except on the proximal ^1^/_3_ and near the lamina bases, where they are present). Furthermore, the narrow habit and imbricate leaves of *S. cyclophylla* resemble those of some liverworts from the genus *Porella* L. [e.g., *P. navicularis* (Lehm. & Lindenb.) Pfeiff. and *P. obtusata* (Taylor) Trevis.].

The type collection of *S. rhodostachya* is based on *im Thurn s.n*., but as Baker (1886: 211) noted, the parentheses around the number (271*) in the protologue refer to the species’ position in the sequence of the authors’ “Synopsis Filicum”. In fact, “*im Thurn 271*” corresponds to *Trichomanes macilentum* Bosch (see Baker, 1886: 212). Furthermore, E.F. im Thurn (1887) published a verbatim reproduction of his “Notes on the plants observed during the Roraima expedition of 1884,” which Baker also treated, and reiterated the previous information (Baker, 1887b: 288 and 296). Additionally, Baker (1887a: 112), referencing his earlier publication (i.e., Baker 1887b), listed the type of *S. rhodostachya* as “*im Thurn s.n*.” It is worth noting that in Baker (1887a: 112), *S. rhodostachya* is listed as species number “292” rather than “(271*)”. Therefore, the correct type citation for *S. rhodostachya* should be “*im Thurn s.n*.”

Furthermore, an isotype of *S. rhodostachya* at BM is labeled “*im Thurn 226*,” but this is also likely a mistake, as the type collection was not numbered, and the number “226” actually refers to the type collection of *S. vernicosa* Baker (Baker, 1886: 220). It seems that whoever created this label unintentionally misnumbered it. This is clear because a) the holotype at K lacks an original label and only has a later note by an unknown source; b) the isotype label at US differs from that at K, which was initially numbered in pencil as “227,” then crossed out and replaced with “271” in pencil, matching Baker’s numbering system; and c) there is a penciled note on the isotype label at BM, probably by Alston, indicating that “271” is wrong. Therefore, it’s reasonable to assume that the specimens labeled “226,” “227,” and “271” at BM and “271” at US are mislabeled, mostly corresponding to the species’ position or number in the sequence of Baker’s “Synopsis Filicum”. Accordingly, “227,” and “271” are considered duplicates of the original collection of *S. rhodostachya* (i.e., *im Thurn s.n*.) and, thus, isotypes. Sadly, this was likely missed by Alston et al. (1981: 297), probably because the first author’s original work on *Selaginella* was completed many years after his untimely death in 1958 (see Alston et al. 1981: 233–236).

A similar confusion exists regarding the correct collection number for the type collection of *S. valdepilosa* subsp. *tricholoma*. The label on the holotype reads “*J.A. Steyermark 58016*,” but this appears to be a mistake for *58916*. The label attached to the holotype was typed with a typewriter likely at and sent by the US, while the labels attached to the isotypes at F, GH, MICH, MO, and US are printed (except for the label on a paratype at US, *58916 bis* [a handwritten “9” superimposed on a typed “0”, which is almost identical to the one on the holotype]) and that also represents *S. valdepilosa* subsp. *tricholoma*. Originally, it seemed that there were two similar collection numbers presumably made on the same day, with a gap of “900 numbers” between them, which represent the same new subspecies, but this is unlikely. Indeed, after the revision of Steyermark’s collection book at MO, it was determined that the collection number *Steyermark 58016* corresponds to an angiosperm rather than a *Selaginella* (R.C. Moran and B.K. Holst, pers. com.). Therefore, it is most likely that, when the label for the specimen sent to the British Museum for Alston’s study was typed, a “0” was entered instead of a “9.” Accordingly, it is concluded that the true collection number for the type collection of *S. valdepilosa* subsp. *tricholoma* should be “*58916*” rather than “*58016*”.

The isotypes at F, GH!, MICH!, MO!, NY!, and S! are mixed collections, which we separated and identified as follows: *a* = *S. rhodostachya* and *b* = *S. vernicosa* (Alston previously classified them this way). Additionally, *J.A. Steyermark 58753* at F! was determined by us to be *a* = *S. rhodostachya* and *b* = *S. vernicosa*, while *Tate 466* at NY! is also a mixed collection, which we identified as *a* = *S. vernicosa* and *b* = *S. rhodostachya*.

Finally, other collections (Guyana: Forest path to the top of Kaieteur Fall, Oct 1888, [*G.S*.] *Jenman s.n*., NY!; Kaieteur, Oct 1888, [*G.S*.] *Jenman s.n*., NY!) cited by Alston et al. (1981) under *S. rhodostachya* actually refer to *S. sandwithii* Alston.

## Supplementary Material

XML Treatment for Selaginella brachyclada

XML Treatment for Selaginella rhodostachya

## Additional information

### Artificial Intelligence (AI) use

The authors accept full responsibility for the content of the manuscript, including the disclosure of any use of AI.

No AI tools were used in the preparation of this manuscript.

### Author ORCIDs

I.A. Valdespino https://orcid.org/0000-0003-1004-8289

C.A. López https://orcid.org/0000-0003-1127-8721

### Data availability

All of the data that support the findings of this study are available in the main text.
